# Early-onset colorectal cancer in patients under 30: a case series from Tanzania highlighting an emerging epidemiological shift

**DOI:** 10.1093/jscr/rjag774

**Published:** 2026-09-01

**Authors:** Mujaheed Suleman, Jay Lodhia, Evance Salvatory Rwomurushaka, Felister Uisso, Alex Mremi

**Affiliations:** Department of General Surgery, Kilimanjaro Christian Medical Centrer PO Box 3010, Moshi, Tanzania; Department of General Surgery, NSK Hospital, PO Box 3114, Arusha, Tanzania; Department of General Surgery, Kilimanjaro Christian Medical Centrer PO Box 3010, Moshi, Tanzania; School of Medicine, KCMC University, PO Box 2240, Moshi, Tanzania; Department of General Surgery, Goulburn Base Hospital, Goulburn, NSW, Australia; Department of General Surgery, Kilimanjaro Christian Medical Centrer PO Box 3010, Moshi, Tanzania; Department of Anatomy and Neuroscience, KCMC University, PO Box 2240, Moshi, Tanzania; Department of Radiology, Kilimanjaro Christian Medical Centre, PO Box 3010, Moshi, Tanzania; School of Medicine, KCMC University, PO Box 2240, Moshi, Tanzania; Department of Pathology, Kilimanjaro Christian Medical Centre, PO Box 3010, Moshi, Tanzania; Department of Research and Training, Kilimanjaro Clinical Research Institute, PO Box 2236, Moshi, Tanzania

**Keywords:** early-onset colorectal cancer, mucinous adenocarcinoma, young adults, Tanzania, late presentation

## Abstract

Early-onset colorectal cancer (EOCRC) is increasingly recognized, particularly in low- and middle-income countries where patients often present with advanced disease. We describe a case series of three patients under 30 years managed at a tertiary referral centre in northern Tanzania. All presented after prolonged symptoms, including rectal bleeding, altered bowel habits, abdominal pain, and weight loss. Two patients had rectosigmoid tumors, while one had a right-sided lesion. Histopathology demonstrated mucinous adenocarcinoma in two cases and adenocarcinoma not otherwise specified in one case. One patient had a family history suggestive of hereditary predisposition. Management included surgical intervention, frequently with stoma formation, and chemotherapy where feasible. Outcomes were poor, including one postoperative mortality and one case requiring palliative chemotherapy. Delayed presentation, limited diagnostic resources, and restricted oncology services significantly impacted care. EOCRC in this setting demonstrates aggressive disease and poor prognosis, highlighting the need for early diagnosis and improved cancer care infrastructure.

## Introduction

Colorectal cancer (CRC) is the third most commonly diagnosed malignancy worldwide and the second leading cause of cancer-related mortality, with the global burden projected to reach 2.2 million new cases and 1.1 million deaths annually by 2030 [1, 2]. Traditionally considered a disease of older adults, recent epidemiological data demonstrate a concerning rise in early-onset colorectal cancer (EOCRC), particularly among individuals younger than 30 years, including those in developing countries [1–5]. This shift is thought to be multifactorial, involving hereditary cancer syndromes, obesity, dietary transitions, increasing alcohol consumption, sedentary lifestyles associated with urbanization, and limited access to screening and early diagnostic services [2, 3, 6, 7].

Estimates of CC incidence in Africa are still not representative of the actual burden of the disease, mainly due to a lack of adequate infrastructure to diagnose patients with CRC, and a lack of population-based cancer registries [8]. In this case series, we present three patients under the age of 30 years with CRC, their outcomes, and challenges faced in a limited-resource setting.

## Cases presentation

### Case 1

A 19-year-old male with no known comorbidities presented with a 3-week history of progressive abdominal distension, abdominal pain, constipation but passing flatus, early satiety, and nausea without vomiting. He also reported a 5-month history of altered bowel habits characterized by alternating diarrhoea and constipation, along with three recent episodes of mucoid, blood-stained stools. This was accompanied by significant unintentional weight loss, easy fatigability, and exertional dyspnoea. There was no family history of malignancy, nor history of smoking or alcohol use.

On examination, he was conscious, afebrile, and not dyspnoeic, but had moderate conjunctival and palmar pallor and bilateral lower limb oedema. Vital signs were stable. His estimated body mass index (BMI) was 19 kg/m^2^. The abdomen was markedly distended, soft, non-tender, hyper-tympanic, with exaggerated bowel sounds. Digital rectal examination revealed a hard, friable rectal mass that bled on contact and completely obstructed the lumen. Chest examination demonstrated reduced air entry in the left mid and lower zones.

Laboratory investigations showed anaemia (Hb 9.4 g/dl), with normal leukocyte and platelet counts. Renal function was normal (creatinine 34 μmol/L and estimated glomerular filtration rate of >90), with mild hyponatraemia (131 mmol/L) and hypokalaemia (3.0 mmol/L).

**Figure 1:**
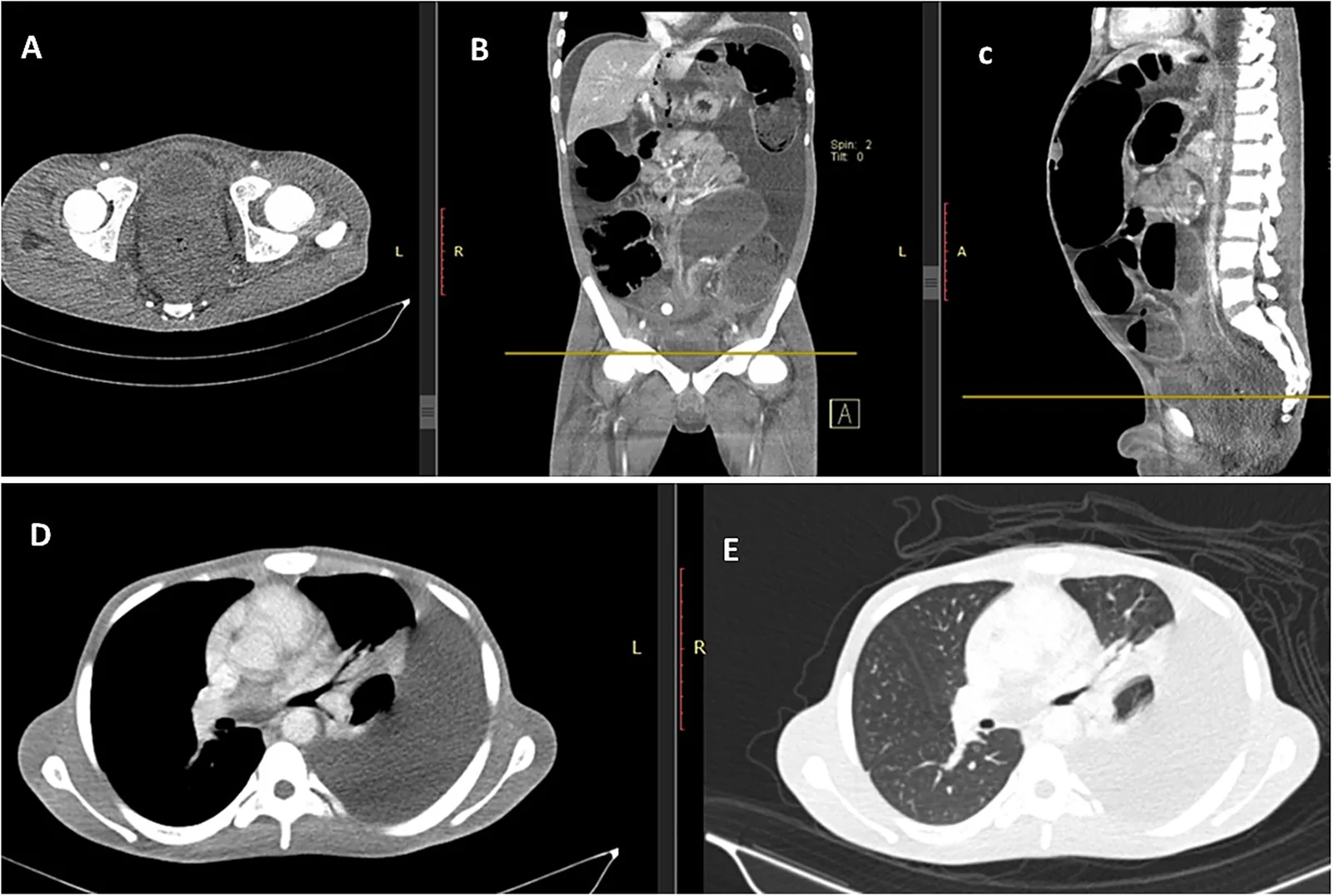
Contrasted abdominal-pelvic CT scan images; A (axial), B (coronal) and C (sagittal) planes shows marked recto-sigmoid stenosing symmetrical mural wall thickening of 4.2 and 16 cm in length with subsequent upstream large bowel obstruction. Small volume pneumoperitoneum suggesting tumor perforation, and moderate ascites noted. D (mediastinal window) and E (lung window) depicts severe left pleural effusion with left lower lobe compressive atelectasis. No metastatic pulmonary nodules.

Contrast-enhanced CT of the chest and abdomen demonstrated marked large bowel dilatation with a transition point at the sigmoid colon, multiple air–fluid levels (largest 9.2 cm), and circumferential rectosigmoid mural thickening (4.2 cm thickness over 16 cm length), with pneumoperitoneum—suggestive of a perforated rectosigmoid tumour. Ascites and a large left pleural effusion with left lower lobe collapse were also noted, raising concern for metastatic disease (Fig. 1).

A chest tube was inserted for pleural drainage, followed by emergency laparotomy after obtaining a written informed consent from the patient and his parents. Intra-operatively, there was grossly dilated, oedematous large bowel with distal obstruction at the tumour site, ~2 litres of serosanguinous ascites, and a hard rectal mass extending to the anus with peritoneal seedlings involving the mesentery, omentum, anterior abdominal wall, and minimally the liver surface. A double-barrel transverse colostomy was fashioned, tumour biopsy obtained transanally, and peritoneal lavage performed.

Postoperatively, the patient initially improved and was discharged on Day 9. However, he developed sudden-onset respiratory distress on the day of discharge and died despite resuscitative efforts; pulmonary embolism was suspected.

**Figure 2:**
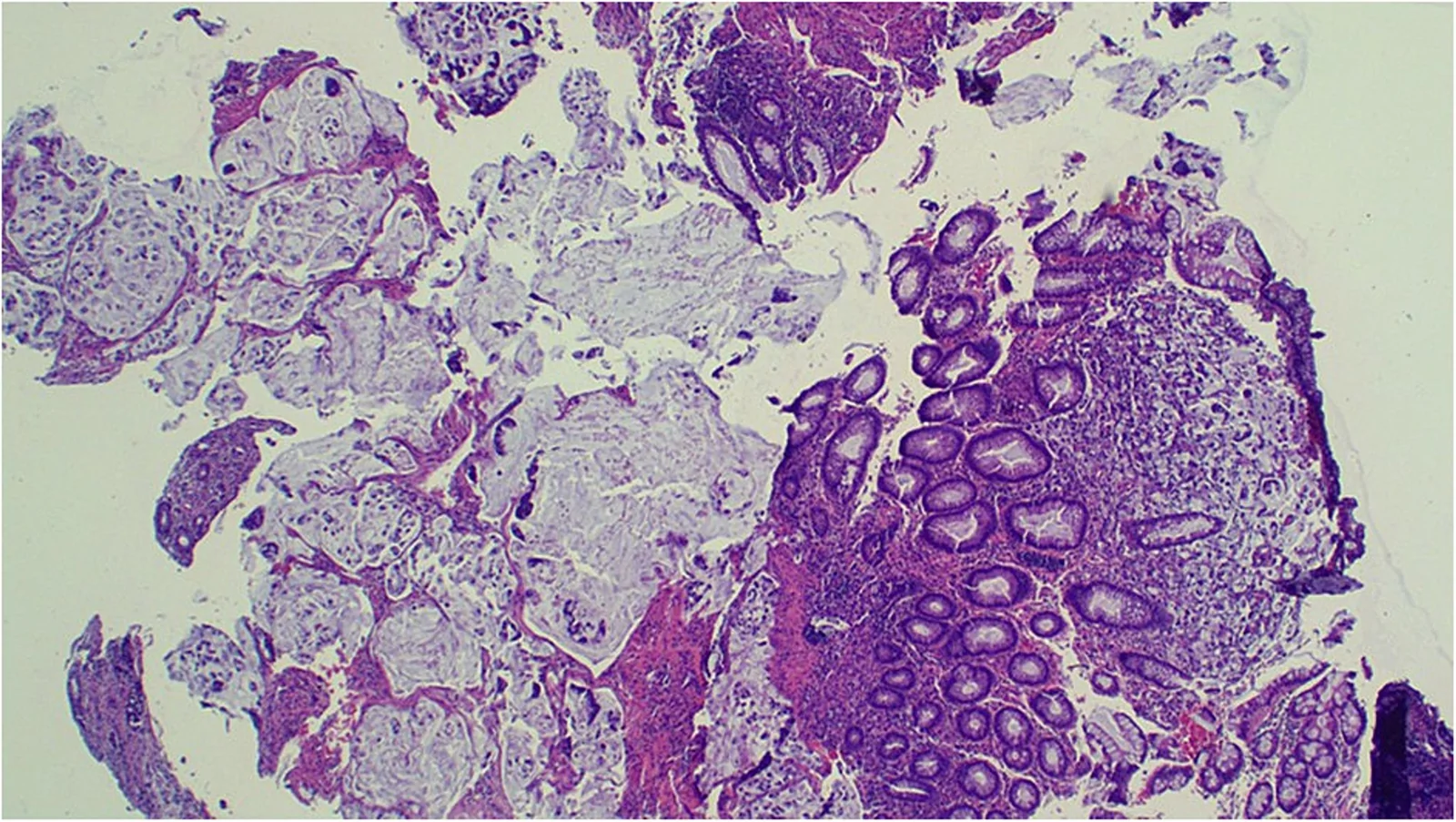
Low-power photomicrograph of mucinous colorectal adenocarcinoma showing abundant extracellular mucin comprising >50% of the tumour, with irregular pools of mucin dissecting through the bowel wall and containing scattered malignant epithelial cells and small clusters with focal gland formation. The neoplastic cells exhibit nuclear atypia and are seen floating within mucin lakes, with associated stromal reaction (×4 magnification).

Histopathology (available posthumously) revealed mucinous adenocarcinoma with omental metastasis (Fig. 2).

### Case 2

A 29-year-old female presented with a 2-month history of abdominal pain and progressive distension, associated with intermittent hematochezia, anaemia, anorexia, weight loss, occasional vomiting, and intermittent fever. She had undergone myomectomy for uterine fibroids three months prior. Notably, she reported a strong family history of CC affecting three maternal relatives.

On examination, she was conscious, afebrile, and mildly pale, with no peripheral lymphadenopathy. Abdominal examination revealed a mobile, tender mass in the right lumbar region.

**Figure 3:**
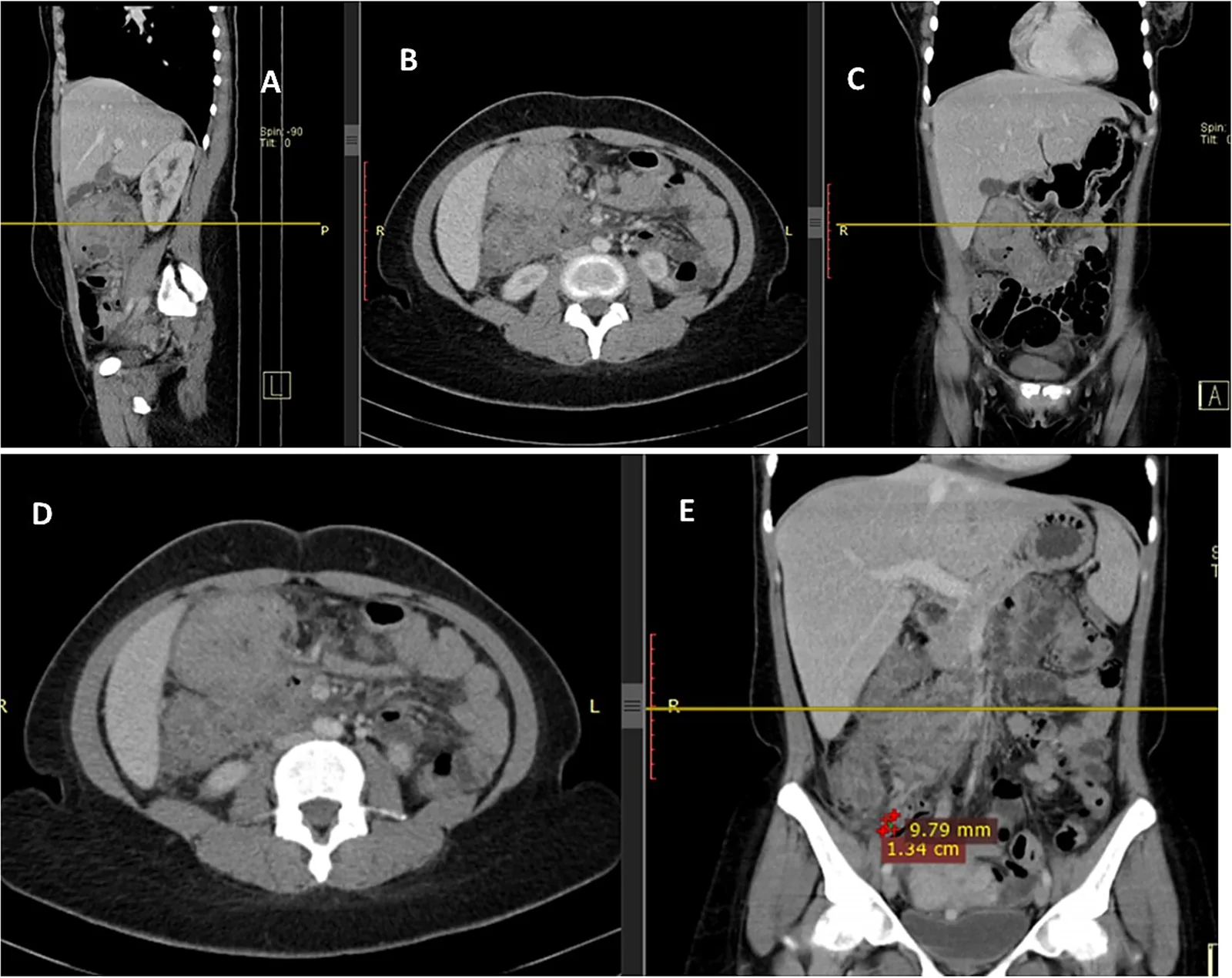
Contrasted abdominal-pelvic CT scan images; A (sagittal), B (axial) and C (coronal) planes showing a large, 7.04 × 5.2 × 8.58 cm sized heterogenous enhancing soft tissue lesion centered at the hepatic flexure abutting the distal D2, extending to both distal ascending colon and proximal transverse colon with perilesional mesenteric fat stranding. Images D (axial) and E (coronal) depicts tumour infiltration to middle colic and right colic branches, as well as abutment of the infrarenal inferior vena cava and inferior mesenteric vein. Multiple perilesional, mesenteric and retroperitoneal lymphadenopathy also noted. There were no pulmonary, hepatic or osseous metastasis noted.

Colonoscopy showed normal distal colonic mucosa; however, the ascending colon and terminal ileum were not visualized due to poor tolerance. Computed tomography (CT) imaging demonstrated a heterogeneous mass at the hepatic flexure involving the distal ascending and proximal transverse colon, with circumferential wall thickening (0.6–1.7 cm), mesenteric fat stranding, and multiple enlarged mesenteric and retroperitoneal lymph nodes. No distant metastases were identified (Fig. 3).

**Figure 4:**
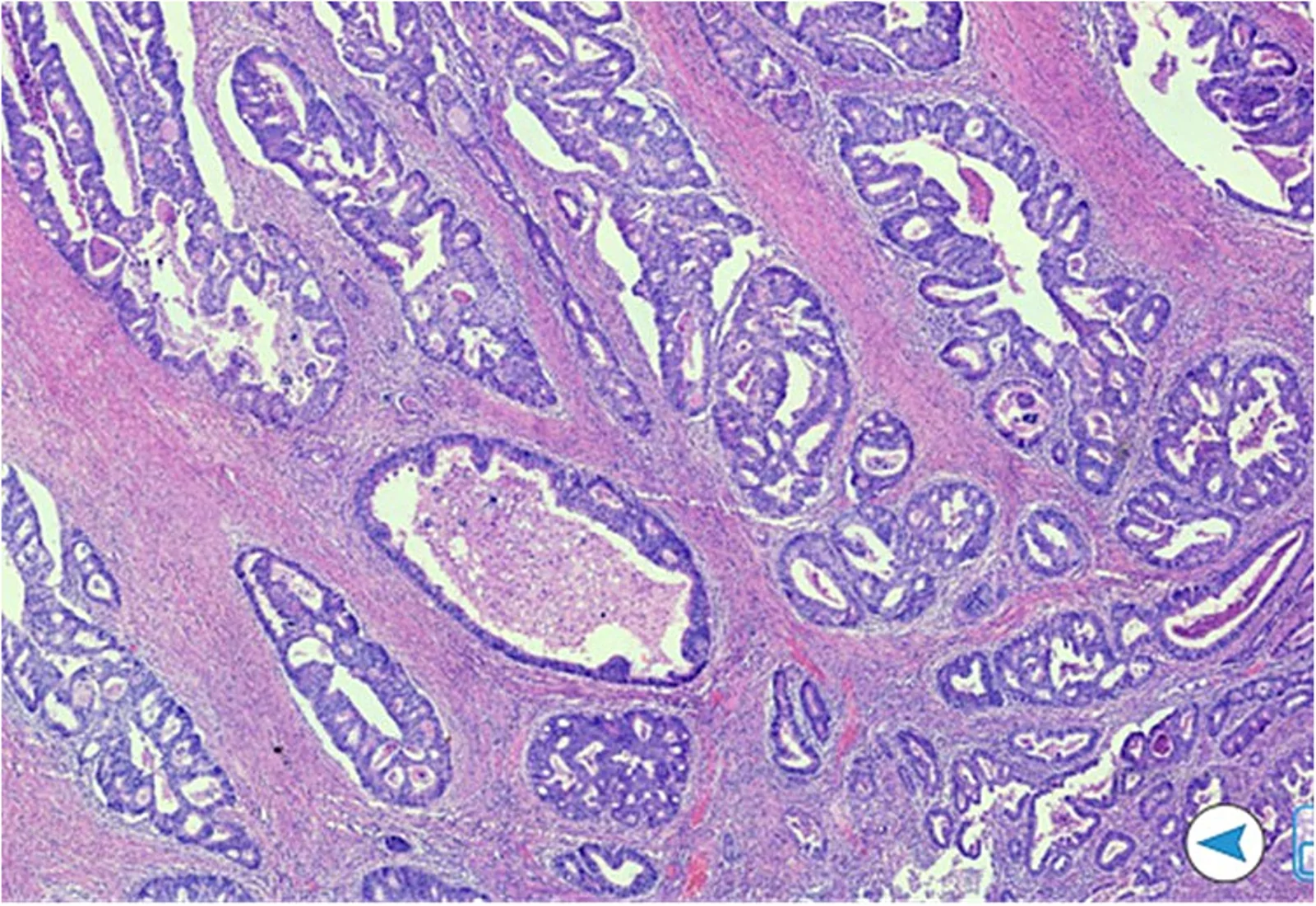
Photomicrograph of colorectal adenocarcinoma showing irregular, infiltrative malignant glands with central comedo-type necrosis characterized by luminal necrotic debris within dilated tumor glands. The surrounding neoplastic epithelium exhibits nuclear atypia and pseudostratification, set within a desmoplastic stroma (×10 magnification).

She underwent extended right hemicolectomy as a semi-urgent procedure and end-to-side ileo-transverse anastomosis. Histopathology confirmed adenocarcinoma of the ascending colon (NOS), staged pT2N1bMx (Fig. 4). Postoperative recovery was uneventful.

At the multidisciplinary tumour board review, adjuvant chemotherapy was recommended. Mismatch repair (MMR) immunohistochemistry was requested but delayed due to a specimen handling error. At follow-up, she remained clinically stable (ECOG 0) and opted to continue treatment at the Ocean Road Cancer Institute. She was recommended undergoing genetic testing.

### Case 3

A 28-year-old male presented with a 2-month history of rectal bleeding and progressive difficulty in defecation, characterized by straining, incomplete rectal emptying, and pencil-thin stools. He denied nausea, vomiting, and had no family history of malignancy or history of smoking or alcohol use.

On examination, he was mildly pale but otherwise stable with a BMI of 23.4 kg/m^2^. Abdominal examination revealed suprapubic tenderness. Digital rectal examination identified a hard, smooth, non-tender mass on the anterior rectal wall, with the examining finger unable to pass beyond it.

**Figure 5:**
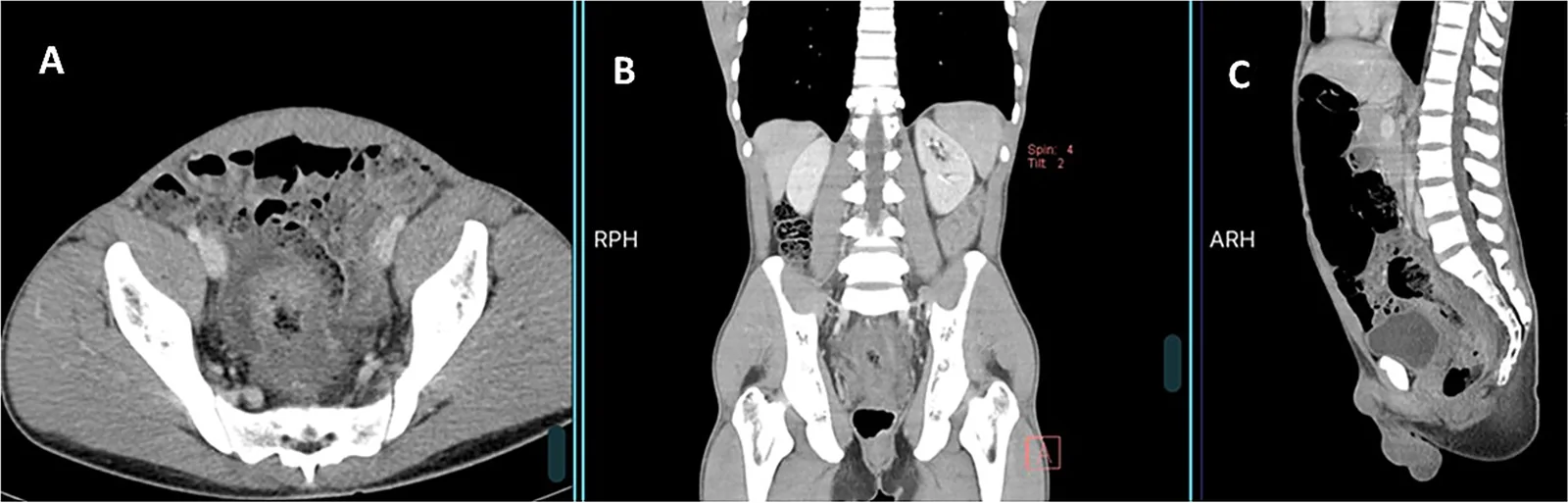
Contrasted abdominal-pelvic CT scan images; A (axial), B (coronal) and C (sagittal) showing a non-stenosing circumferential wall thickening involving the distal sigmoid colon to mid-rectum of 3.12 cm maximum thickness, with surrounding mesorectal fat stranding and subcentimeter lymphnodes. Mild distention of the upstream large bowel loops noted. Mild ascites was present (not shown in the images). No distant metastasis was detected.

Colonoscopy demonstrated a stenosing rectal tumour located 10 cm from the anal verge, preventing further scope advancement. His initial CT imaging revealed circumferential rectosigmoid wall thickening (maximum 3.12 cm) with mesorectal fat stranding, luminal narrowing, and proximal faecal loading with mild colonic dilatation. Small regional lymph nodes were noted without distant metastases (Fig. 5).

**Figure 6:**
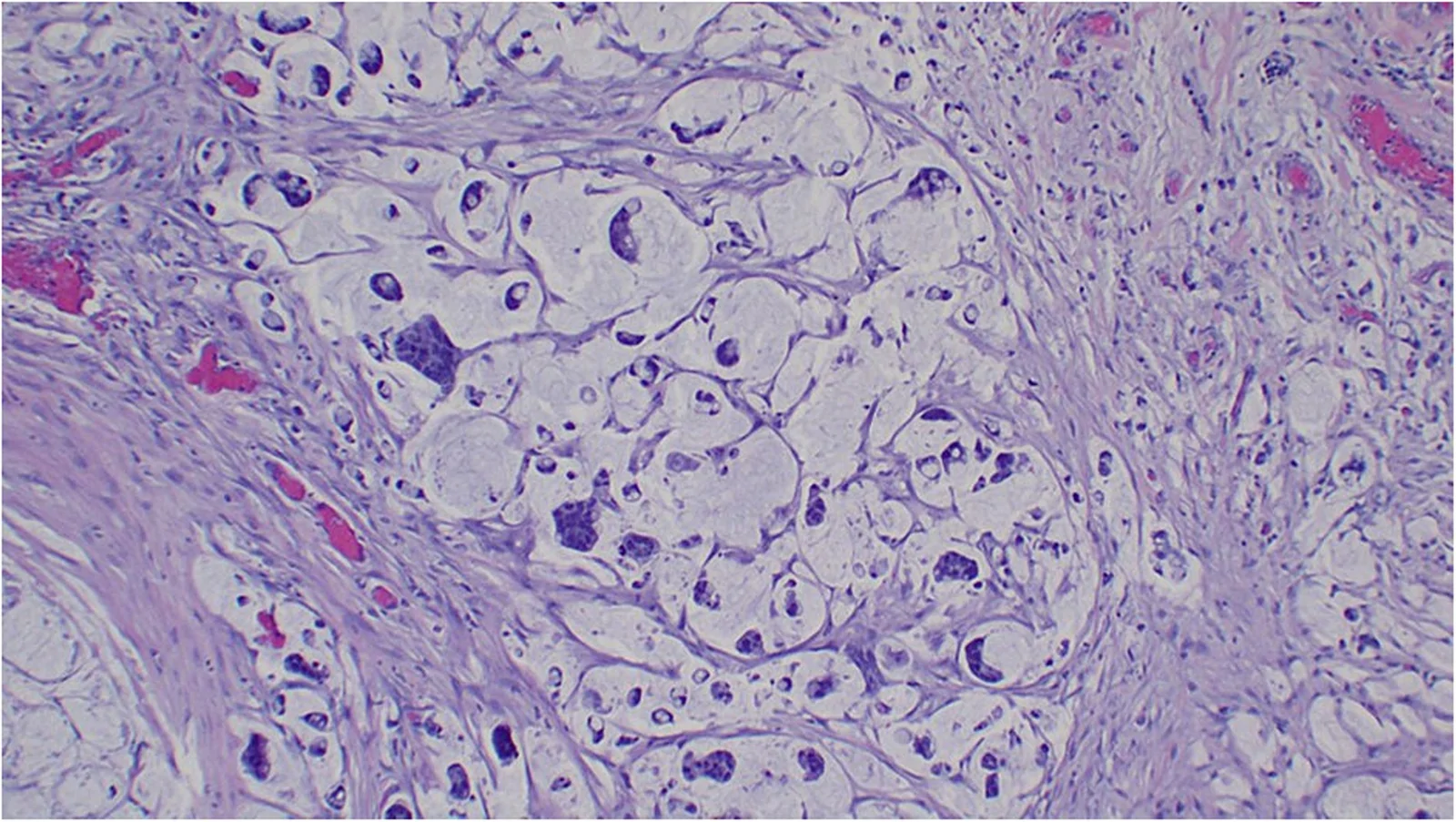
Photomicrograph of mucinous colorectal adenocarcinoma showing abundant extracellular mucin lakes containing clusters and singly dispersed malignant epithelial cells, including signet ring-like forms. The tumor cells exhibit marked nuclear atypia and are suspended within mucin, with focal stromal desmoplasia at the periphery (×20 magnification).

Histopathology confirmed mucinous adenocarcinoma (Fig. 6). The patient was discussed at a multidisciplinary tumour board but unfortunately was lost to follow-up. He re-presented 2 months later with clinical features of peritonitis and radiographic evidence of pneumoperitoneum.

Emergency laparotomy revealed purulent peritoneal fluid, a pelvic abscess, peritoneal seedlings, and a rectosigmoid mass adherent to surrounding structures without frank perforation. A sigmoid loop colostomy was performed, and cultures grew *E. coli* (Escherichia coli) sensitive to amoxicillin–clavulanate. Postoperative recovery was complicated by superficial surgical site infection.

Subsequent imaging showed disease progression with bilateral pleural effusions. Due to metastatic features and resource limitations [magnetic resonance imaging (MRI) not performed], he was commenced on palliative chemotherapy (capecitabine–oxaliplatin). After eight cycles, imaging showed stable disease with resolution of pleural effusion.

## Discussion

This case series highlights the clinical and systemic challenges associated with EOCRC in a resource-limited setting. Although CC has traditionally been considered a disease of older adults, its incidence is increasing among younger populations worldwide, with a disproportionate burden in low- and middle-income countries [6]. Consistent with previous reports, all three patients in our series presented with advanced-stage disease, characterized by obstructive symptoms, substantial tumour burden, and, in some cases, metastatic or disseminated disease [9–11]. Symptom duration ranged from several weeks to months, with rectal bleeding, altered bowel habits, weight loss, and abdominal pain often being overlooked or initially attributed to benign conditions, contributing to delayed diagnosis and limited treatment options.

Histopathologically, two of the three patients had mucinous adenocarcinoma, a subtype that is more common in younger patients and is associated with advanced disease, poorer prognosis, and reduced responsiveness to conventional chemotherapy [12–14]. This supports emerging evidence that EOCRC may exhibit distinct biological characteristics compared with late-onset disease [10]. Molecular characterization was limited by restricted access to MMR protein analysis and microsatellite instability testing. Furthermore, one patient had a strong family history of colorectal cancer, raising suspicion for an underlying hereditary cancer syndrome, such as Lynch syndrome, although genetic testing was not available.

Delays in diagnosis and treatment reflected broader health system constraints. Limited access to colonoscopy, advanced imaging, timely histopathology, and immunohistochemistry contributed to delayed staging and initiation of treatment. Financial barriers prevented MRI in one patient, while specimen handling issues delayed immunohistochemistry in another, highlighting the need to strengthen diagnostic infrastructure and pathology services in resource-limited settings [3]. Although colonoscopy remains the gold standard for colorectal cancer diagnosis and screening, widespread implementation may not currently be feasible in many low-resource settings. A phased approach incorporating affordable strategies, such as faecal immunochemical testing, selective flexible sigmoidoscopy, and targeted screening of high-risk individuals, may represent a more practical pathway while endoscopic capacity continues to develop. Furthermore, the increasing availability of CT scanners in Tanzania provides an opportunity to facilitate earlier detection and staging of suspected colorectal malignancies within existing healthcare infrastructure [15].

Management was primarily determined by advanced disease at presentation and available resources. Most patients required emergency or palliative surgical procedures, including diversion colostomies for obstruction or perforation, with treatment decisions guided through multidisciplinary discussions where feasible. Despite these efforts, outcomes remained poor: one patient died postoperatively, likely from pulmonary embolism, while another experienced disease progression despite chemotherapy, underscoring the combined impact of aggressive tumour biology, delayed presentation, and limited access to comprehensive oncological care [16].

These findings have important implications for clinical practice and public health in Tanzania. Clinicians should maintain a low threshold for investigating alarm symptoms such as rectal bleeding, altered bowel habits, unexplained anaemia, and weight loss in younger patients to minimize delays resulting from age-related diagnostic bias. Strengthening diagnostic capacity through timely histopathology, access to immunohistochemistry, and multidisciplinary cancer care is essential for accurate diagnosis and treatment planning [17–19]. Given that ~70% of Tanzania’s population comprises individuals aged 15–64 years, the rising burden of early-onset colorectal cancer threatens both population health and national productivity [20]. Increasing public awareness, promoting timely health-seeking behaviour, and implementing context-appropriate screening and early detection strategies, including improved access to colonoscopy for high-risk and symptomatic individuals, are critical to improving outcomes in this young population.

The main limitation of this report is the small sample size inherent to a case series, which limits generalizability. However, the detailed clinical and pathological descriptions provide valuable insights into the presentation and management of EOCRC in a low-resource setting. Given the observed trends and the availability of additional cases within our institution, future studies with larger cohorts are warranted to better define the epidemiological and molecular characteristics of EOCRC in this population.

Early-onset colorectal cancer in patients <30 years remains an uncommon but increasingly recognized entity in sub-Saharan Africa, often presenting at an advanced stage with aggressive histopathological features and poor clinical outcomes. Delayed diagnosis, driven by low clinical suspicion, nonspecific symptoms, limited access to endoscopy and pathology services, and poor health-seeking behaviour, continues to be a major challenge. Clinicians should maintain a high index of suspicion when young patients present with alarm symptoms such as rectal bleeding, altered bowel habits, unexplained anaemia, or weight loss. Improving public awareness of colorectal cancer symptoms, promoting timely health-seeking behaviour, and expanding access to colonoscopy-based screening and early diagnostic services are essential to facilitate earlier diagnosis. Strengthening multidisciplinary care, improving diagnostic capacity, and implementing context-appropriate screening strategies will be critical to reducing the growing burden of early-onset colorectal cancer and improving patient outcomes in resource-limited settings.

## Data Availability

There are no new data associated with this article.
